# Saudi Expert Consensus-Based Autism Spectrum Disorder Statement: From Screening to Management

**DOI:** 10.3390/children9091269

**Published:** 2022-08-23

**Authors:** Shuliweeh Alenezi, Ahmad S. Alyahya, Shahad M. AlKhalifah, Hadeel R. Bakhsh, Eiman H. Alismail, Hesham Aldhalaan, Talat Alwazna, Nouf Alzrayer, Saleh S. AlSuwailem, Faisal Alnemary, Ahmed M. S. AlAnsari, Enas I. Alqulaq, Amal Alyamani, Yasser S. Amer, Ibrahim M. Albawardi, Waled M. Albalawi, Mohammed A. Alhassan, Maha S. Algazlan, Malak Alramady, Yasser Ad-Dab’bagh

**Affiliations:** 1 Department of Psychiatry, College of Medicine, King Saud University, Riyadh 11451, Saudi Arabia; 2 Department of Psychiatry, Eradah Complex for Mental Health, Riyadh 12571, Saudi Arabia; 3 Center for Autism Research, King Faisal Specialist Hospital & Research Center, Riyadh 11564, Saudi Arabia; 4 Department of Rehabilitation, College of Health and Rehabilitation Sciences, Princess Nourah Bint Abdulrahman University, Riyadh 11564, Saudi Arabia; 5 Department of Neuroscience, King Faisal Specialist Hospital & Research Center, Riyadh 11564, Saudi Arabia; 6 Department of Neurology, King Saud Medical City, Riyadh 12746, Saudi Arabia; 7 Department of Special Education, College of Education, King Saud University, Riyadh 11451, Saudi Arabia; 8 Autism Center of Excellence, Riyadh 11564, Saudi Arabia; 9 Department of Psychiatry, College of Medecine and Medical Sciences, Arabian Gulf University, Manama 293, Bahrain; 10 Prince Mohammed Bin Salman Center for Autism and Neurodevelopment, Riyadh 12426, Saudi Arabia; 11 Health Services of Ministry of Defense, Riyadh 12426, Saudi Arabia; 12 Department of Psychiatry, King Fahad Armed Forces Hospital, Jeddah 23311, Saudi Arabia; 13 Pediatrics Department, King Khalid University Hospital, Riyadh 12372, Saudi Arabia; 14 Quality Management Department, King Saud University Medical City, Riyadh 12746, Saudi Arabia; 15 Evidence-Based Health Care and Knowledge Translation, King Saud University, Riyadh 11451, Saudi Arabia; 16 Alexandria Center for Evidence-Based Clinical Practice Guidelines, Alexandria University, Alexandria 5424041, Egypt; 17 Adaptation Working Group, Guidelines International Network (GIN), Perth PH16 5BU, UK; 18 Department of Psychiatry, College of Medicine, Imam Abdulrahman bin Faisal University, Dammam 34212, Saudi Arabia; 19 King Salman Armed Forces Hospital, Tabuk 47512, Saudi Arabia; 20 Mental Health Department, Neuroscience Center, King Fahad Specialist Hospital-Dammam (KFSH-D), Dammam 32253, Saudi Arabia

**Keywords:** autism spectrum disorder (ASD), guideline, assessment, intervention, monitoring

## Abstract

Background: There is a large gap between the needs of individuals diagnosed with autism spectrum disorder (ASD) and the currently available services in Saudi Arabia. Services are often difficult to access, inconsistent in quality, incomplete, unsatisfactory, and costly. As such, there is a national need for expert consensus on the appropriate standards for the assessment and management of children on the autism spectrum. Methodology: A guideline development group (GDC) was formed by professionals representing all related specialties and institutions involved in the management of individuals on the autism spectrum in Saudi Arabia. They met on a regular basis over 21 months. The guideline development process consisted of five steps starting from reviewing existing guidelines and ending with discussing and writing this manuscript. A formal voting process was utilized and recommendations were discussed until a consensus was reached. Results: There was consensus on the following: A specialized diagnostic assessment needs to be carried out by an experienced multidisciplinary team for children referred to assess for ASD. They should be assessed for medical etiology, their behavioral history carefully reviewed, and symptoms directly observed. Longitudinal assessments are encouraged to reflect the effects of symptoms on the individual’s ability to function while with their family, among peers, and in school settings. An additional formal assessment of language, cognitive, and adaptive abilities as well as sensory status is essential to complete the diagnostic process. Interventions should be individualized, developmentally appropriate, and intensive, with performance data relevant to intervention goals to evaluate and adjust interventions. Target symptoms must be identified to address and develop monitoring systems to track change. Conclusion: ASD is a complex condition with widely varying clinical manifestations, thus requiring evaluation and intervention by a range of professionals working in coordination. Behavioral and environmental interventions are the key to optimal outcomes, in conjunction with medications when indicated for specific symptoms. Parental involvement in interventions is vital to sustaining therapeutic gains.

## 1. Introduction

Autism spectrum disorder (ASD) is a neurodevelopmental disorder with onset in early childhood. A diagnosis of ASD requires the presence of impairment in two major domains: communication and social interaction, and restricted or repetitive patterns of behavior [1]. ASD is considered multifactorial in origin where multiple genetic and epigenetic factors may contribute to the etiology [2]. Research on ASD is relatively limited in developing countries, including Saudi Arabia, and thus most of the current clinical practices in Saudi Arabia are based on conclusions from studies conducted in Western developed countries [3]. Although a marked growth in local research started to emerge in 2008, there is much that still has to be investigated. For instance, the prevalence of ASD in Saudi Arabia has not yet been accurately determined. One study stated that 42,500 cases were diagnosed with autism in 2002 [4]. A more recent systematic review showed that the prevalence of ASD in Arabian Gulf countries, including Saudi Arabia, ranged from 1.4 to 29 per 10,000 population [5]. This is, in fact, lower than the prevalence rate measured by studies conducted in developed countries (39–77 per 10,000). This does not necessarily mean that it is less prevalent in Saudi Arabia, as the discrepancy could be explained by methodological flaws, the limited capacity to diagnose ASD, and lower level of awareness of ASD among parents that reduces the likelihood of recognizing symptoms and consequent attempts to access care [6]. A cross-sectional study involving 205 individuals with ASD showed that the ratio between males to female in that sample was 4.9:1. Psychiatric comorbidities were found in 65% of the patients. Among these comorbidities, attention deficit hyperactivity disorder (ADHD) was the most prevalent (53%), followed by intellectual disability (8%), then epilepsy and cerebral palsy (2% for each) [7]. 

In Saudi Arabia, there is a considerable gap between the needs of individuals diagnosed with ASD and the currently available services. Although Saudi Arabia is a large country, most of the services are localized in its largest cities (i.e., Riyadh, Jeddah, or Dammam) [7]. Furthermore, the provided services tend not to meet the satisfaction of families. In Saudi, a study that used a scale of excellent–good–acceptable–bad to evaluate publicly provided services for ASD showed that about 75% of the families scored them as acceptable or bad [7]. Attesting to difficulties in accessing services in Saudi Arabia, Khan et al., in a recent cross-sectional study published in 2020, found that only 31% of children on the autism spectrum could access nearby autism centers, and 72% have no access to private schools for ASD in their area [8]. The areas that are targeted through early intervention are language, play skills and social participation, activities of daily living (ADLs), and challenging behaviors [9]. Moreover, pharmacological management can be initiated to target specific symptoms related to ASD, such as irritability, hyperactivity, aggression, aberrant social behavior, repetitive behavior, inattention, and insomnia. However, all medications might cause side effects that need to be monitored by a professional, so it should be prescribed by either a neurologist or a psychiatrist [10]. In one study conducted in Saudi Arabia, almost half of the children on the autism spectrum were prescribed psychotropic medications mainly for behavioral management and risperidone was the most prescribed medication [11].

The lack of governmental and institutional support for a child on the autism spectrum places a significant burden on parents, which might affect the quality of life for other siblings. It has been shown that almost 88.5% of parents of children on the autism spectrum in Saudi Arabia experience significant distress. The challenges and unmet needs of children on the autism spectrum resulting from lack of access to needed resources resulted in negative impacts on social and financial aspects of their lives, their family relationships, and the quality of life of the siblings of the children on the autism spectrum [11]. In another cross-sectional study that involved 61 families of individuals diagnosed with ASD, 37% of the families felt embarrassed for having children on the autism spectrum, while almost 64% were bothered by the way that others in the community dealt with them [6].

Currently, several authorities are involved in the journey of care, education, and empowerment for individuals diagnosed with ASD and others with other developmental disabilities. The initial screening (i.e., especially at a young age) and diagnostic services are carried out by specialized and trained healthcare professionals (i.e., general pediatricians, psychiatrists, child psychologists, developmental-behavioral or neurodevelopmental pediatricians, pediatric neurologists, or child psychiatrists) working in entities affiliated with the Ministry of Health (MoH) or accredited by public authorizing entities, such as Saudi Health Council (SHC), Saudi Commission for Health Specialties (SCFHS), and Saudi Central Board of Accreditation of Healthcare Institutions (CBAHI). This discrepancy in carrying out such services with different capacities might be attributed to the lack of clear guidance, shortage of specialized professionals, and availability of validated tools. Therefore, there have been calls to develop a nation-based policy to mitigate the lack of services for children on the autism spectrum [12].

For post-diagnostic services, rehabilitation services are overseen by three entities: the MoH, the Ministry of Education (MoE), and the Ministry of Human Resources and Social Development (MHRSD), where services are provided through governmental (free-of-charge) providers or private (pay-for-services or covered by health insurance) ones (e.g., hospitals, schools, special needs centers, and specialized clinics). Despite some significant and successful initiatives to enhance accessibility and improve the quality of services, more need to be achieved in this regard [13]. While MHRSD is overseeing and subsidizing intervention services that private centers provide to children who are not in school because they are younger than six years old or are severely impacted (IQ > 50), MoE serves students on the autism spectrum that do not have severe intellectual disability and have effective verbal communication in their public school systems, including some institutions that are designated for students with disabilities, special-needs classrooms, or mainstreamed (inclusion) classrooms along with their typical peers. 

Intervention and special education services may vary from one center to another. Generally, the number of intervention hours received and its structure (group-format vs. individualized-format) varies mainly based on the limited service slots, ranging from 20 h/week to only a few. However, about 80% of families of individuals on the autism spectrum accessing early intervention services pay for them out-of-pocket and almost 92% of families reported an excessive financial burden related to the management of the needs of their children on the autism spectrum [6]. As such, the status of services in Saudi Arabia might require a thorough review of the needs of individuals on the autism spectrum and their families and concerted efforts to attempt to meet them. To do so, there is a national need for expert consensus on appropriate standards for the assessment and management of children on the autism spectrum. This became possible after the Prince Mohammed Bin Salman Program for Autism and Developmental Disorders was established in 2018, which took this role to help to address those gaps in practice and funded this project to pave the way for more standardized care.

## 2. Materials and Methods

### 2.1. Team of Experts 

The methodological approach utilized was based on a formal voting technique that included the review of the available scientific evidence and the involvement of ASD clinical experts to develop consensus-based recommendations on screening, assessment, and interventions for ASD in children under 16 years of age.

The expert consensus development process was conducted between 12/2018 and 09/2020 by a multidisciplinary expert clinical panel and a guideline development group (GDG) of five physicians (three child psychiatrists, a pediatric neurologist, and an adult neurologist) and ten doctoral level experts from relevant specialties (two clinical psychologists, two behavioral therapists, two speech and language pathologists, two occupational therapists, and two special education experts) from different healthcare-providing sectors that contribute to the healthcare provision for individuals diagnosed with ASD.

### 2.2. Guideline Development and Process 

The process consisted of five steps: (i) literature review of the existing international guidelines; (ii) formulation of subgroups divided by clinical specialty to be assigned a relevant section of ASD management; (iii) each subgroup developed an initial draft of recommendations relevant to their specialty; (iv) review and discussion of the first draft from each subgroup with the larger group for integration, continuity of care, and finalization; and (v) writing up the final draft as a comprehensive clinical practice guideline (CPG) adapted to suit the Saudi Arabian culture and current healthcare system and having it vetted by a quality and patient safety team. 

In the first step, a literature review was conducted to identify published evidence and practice guidelines relevant to the screening, diagnosis, intervention, and management of children with ASD. In the second and third steps, based on the results of the literature review and opinions of the assigned specialized subgroups, an initial draft of their specific recommendations was prepared by each of the clinical specialties. In the fourth step, each of the initial drafts was presented to the larger group to adjust, when necessary, multidisciplinarity and integration of care of children with ASD and to eliminate redundancies. In the final step, the final draft of the CPG’s recommendations was reviewed and approved using the Continuous Quality Improvement and Patient Safety Team (CQIPST) at Prince Sultan Military Medical City (PSMMC). The values and preferences of the child, parents, and caregivers as relevant to the Saudi Arabian context were considered during the finalization of the recommendations.

Overall, the GDG and its subgroups met on a regular basis for over 21 months, conducting 45 sessions; all were face-to-face meetings, except for the last two that were conducted online in compliance with precautionary measures related to the COVID-19 pandemic. The chair of the GDG was responsible to facilitate the discussions required to address and resolve any disagreements during these meetings for each recommendation statement by using formal voting.

A set of CPG implementation tools was incorporated in the finalized CPG document to facilitate future implementability. These tools included: (i) a method for the identification of the most responsible physician, (ii) a list of minimum standard qualifications for each category of healthcare providers that contributes to the healthcare provision, (iii) providers’ clinical documentation sheets, and (iv) four key performance indicators (KPIs) to support adherence to the CPG recommendations and audit and feedback. The full CPG document was finalized and approved with input from members of the GDG who contributed to the creation of new national accreditation standards for ASD centers by the Saudi Central Board for Accreditation of Healthcare Institutions (CBAHI).

## 3. Results

Table 1, Table 2, Table 3, Table 4 and Table 5 below show key recommendations divided into different phases of management. 

## 4. Discussion

This is the first national set of clinical practice recommendations that should serve as a foundation stone in standardizing the assessment, management, and monitoring delivered to individuals on the autism spectrum in Saudi Arabia. The early identification and intervention of children with ASD are emphasized in our recommendations and follow multiple international guidelines. Although our recommendations did not provide key signs for identifying ASD, we relied on physician’s clinical judgement and on the utilization of the DSM-5 criteria to guide the diagnostic process [15]. However, it was interesting to see that only the New Zealand’s and National Institute for Health and Care Excellence (NICE) guidelines took the effort and added red flag signs for identification [16,17].

Our recommendations advocate for a proper assessment of the qualification of specialists with direct interaction with such a population. This is particularly important in ABA-based interventions as evidence suggests that variables related to supervision also significantly contribute to treatment outcomes [18]. It also aims to prevent the inconsistency in health professionals’ diagnostic practices and limit the qualification to diagnose such disorders to specific specialties. Nonetheless, the diversity and variety of such proposed specialties would make it more feasible for individuals in rural areas to receive appropriate care. However, many international guidelines (e.g., those from New Zealand and the US) have not specified qualifications or list specialties [16,19,20]. The approaches and interventions of interdisciplinary teams were the emphasis of these consensus recommendations. Another subject covered by our recommendations and those from the United States and Dubai is the need to conduct a comprehensive physical examination during the developmental period, including screening for neurocutaneous disorders, dysmorphic features, and visual and hearing impairments [20,21].

Our recommendations suggest performing genetic evaluation for all individuals with a confirmed ASD diagnosis. Moreover, it advises conducting a chromosomal microarray analysis (CMA) if the etiology of developmental disability is unknown due to the high level of genetic variation and de novo mutations seen in 20–30% of individuals on the autism spectrum [22]. These recommendations took into account the significance of consanguinity in Saudi culture, which was reported at one time to be present in 57.7% of marriages [23]. This differs from the NICE and Dubai’s guidelines, which limit the recommendation for genetic evaluations to when they are deemed clinically indicated [17,21,24].

Contrary to New Zealand’s guidelines, which recommends looking for other comorbidities only when a severe behavioral disturbance is evident, comorbid screening is recommended in our recommendations as well as in the NICE, and European’s for all cases suggestive of ASD [16,17,25]. Although the US guidelines suggest that ASD might mask and overshadow other comorbidities, it did not recommend looking for specific diseases [20]. Similarly, it is critical to recognize these medical issues because many of them can promote or exacerbate undesired behavior in children on the autism spectrum, and these behaviors may improve once the medical issues are managed. On a different note, the telehealth assessment of ASD has been proposed in Australian guidelines; they regulate its role and use, limiting its use but ensuring better care quality [19]. Our recommendations did not address telehealth use as it is conceptually new in our country and is yet to be adequately regulated. However, telehealth initiatives were implemented recently by the MoH, which will facilitate providing and developing such services to individuals on the autism spectrum.

These consensus recommendations lack a clear pathway for the referring physician for a diagnostic assessment, unlike the guidelines from Australia and Dubai, which provided specific instructions for the referring and receiving healthcare providers [19,21]. One report that discussed this matter in New Zealand mentioned that a diagnostic pathway might complicate the process of seeking help from parents, and nearly a quarter of parents were guided down paths that delayed the eventual ASD diagnosis [16,26]. This highlights the importance of implementation tools and their utilization when dealing with a disorder requiring a multidisciplinary approach to its diagnosis. 

In terms of medical interventions, we recommend that the intervention should be individualized, developmentally appropriate, and intensive. Our recommendations indicate that the accurate diagnosis of coexisting psychiatric conditions guide therapy. Consistent with CPGs such as NICE, Scottish Intercollegiate Guidelines Network (SIGN), Indian and expert opinion guidelines such as Dubai, and British Association of Psychopharmacology (BAP), we recommend against the routine use of medications including antipsychotics to treat the core symptoms of ASD [27,28,29]. We find that medications can be helpful to address irritability, challenging behaviors, and co-occurring symptoms and/or disorders. However, they should be considered only after careful accounting of when the behavior started and what seems to exacerbate it. This is consistent with other guidelines, such as those from NICE and New Zealand [16,17]. The higher use of psychostimulants and antipsychotics is observed in individuals on the autism spectrum in primary health care compared with the general population [30]. We agree with Dubai guidelines that the use of psychotropic medications should be part of a comprehensive intervention approach and not used solely [21]. If a medication is to be prescribed for a specific indication, the prescribing clinician should carefully weigh the potential risks and benefits, which is an agreement point among all guidelines. In addition, the prescribing clinician should understand the indications and contraindications, dosing, potential adverse effects, drug–drug interactions, and monitoring requirements of the medications. We do not recommend specific medications for irritability, sleep, ADHD, or anxiety, which were included in New Zealand guidelines, Indian CPGs, and Dubai expert opinion guidelines [16,21,28]. We do not limit medications to specific choices as long as they are evidence-based for reasons related to the development of our healthcare system and our approach toward an individualized plan of intervention. Alternative interventions, such as exclusion diet, secretin chelation, and hyperbaric oxygen therapy, were beyond the scope of these recommendations.

In terms of psychological interventions, our recommendations placed greater emphasis on family involvement: starting from involving the parents or caregivers in the evaluation session so they can directly observe what occurs in that session and discuss these observations with the psychologist afterward. We emphasized delineating a systematized process for defining the family’s/caregivers’ level of involvement, training duration, and expectations. This was not emphasized in other international guidelines, such as those from New Zealand, Australia, and SIGN [16,19,27]. Consistent with the Dubai expert opinion guidelines, the psychoeducation provided to the parents is vital to ensure that the skills learned are generalized to different settings [31]. In alignment with other guidelines, such as NICE and SIGN, we emphasize the importance of social communication interventions in targeting the core symptoms of autism [17,27]. As for behavioral interventions, in addition to parent-mediated approaches, NDBIs and approaches focused on social–pragmatic and relational skills, we recommend early intensive intervention programs (EIP) for young children aged 2–5 years, which differs from the recommendations of New Zealand’s guidelines, in which we targeted a specific age group for EIP [16]. Given that the strongest evidence of EIP efficacy was found to be with this age group and taking into consideration the substantial limitation in the availability of the resources necessary for such programs in Saudi Arabia, we opted not to recommend such programs for older age groups [32]. In contrast to Dubai guidelines, we did not restrict direct therapy hours of EIP to 20–40 h per week, as well as in consideration of the substantial limitation in the availability of the resources necessary for such programs in Saudi Arabia, and useful programs that may be accessible in our country may provide fewer than 20 h of EIP per week. In addition, we did not enforce requirements related to the international certification of ABA service providers to make it feasible in our society where the availability of such trained personnel is limited. 

Similar to other international guidelines such as SIGN, we recommend occupational therapy interventions that emphasize social engagement and participation, developmental activities, domestic and personal skills, and encourage collaboration with speech and language pathologists (SLPs) and behavioral therapists [27]. One of the strengths of our guidelines is that we demonstrated a model for the occupational therapy settings including equipment necessary to ensure safety, such as mats and swings and time of face-to-face interaction to ensure quality. Compared with the Dubai guideline, and while recommending the assessment and management of sensory issues, our guidelines did not specifically recommend sensory-oriented interventions such sensory integration therapy and touch therapy/massage in the management of ASD in children and adolescents due to the lack of solid evidence [33]. In addition to occupational therapy interventions for children with ASD, we also recommended speech and language therapy for the behavioral management of feeding. We elaborated further on the facilitation of activities and participation by helping the individual acquire new communication skills or modify existing skills in addition to the utilization of family-centered practice, which was not discussed in other international guidelines such as NICE and the Indian guidelines, which is another noted strength in our recommendations [17,28,29]. There are current efforts to draft the first national ASD Clinical Practice Guideline (ASD-CPG) using AGREE II Instrument and GRADE Method [34]. The new guideline will take time to develop, and we hope these recommendations will bridge the gap until it is released.

## 5. Conclusions

Considering that ASD is a complex syndrome with a wide range of clinical presentations, it necessitates examination and intervention by a team of clinicians. Behavioral and environmental interventions, in addition to the occasional need for medications, are central to achieve the best results. Parental involvement is vital during the intervention to maintain therapeutic gain. Our expert consensus clinical practice recommendations were developed around these core concepts and it is hoped that they pave the way toward a higher standard of care delivered to children on the autism spectrum in Saudi Arabia.

## Figures and Tables

**Table 1:** Description of the CPG-targeted population and its intended users.

Disease/Condition	Autism Spectrum Disorder (ASD)
**Target Population**	Children under the age of 16
**Clinical Specialty (Intended Users)**	Pediatrics. Family Medicine Physicians. Child and Adolescent Psychiatry. Neurodevelopmental Pediatrics. Pediatric Neurology. Clinical Psychology. Occupational Therapy. Speech and Language Pathology. Special Education. Nursing.
**Recommendations**	
**Multidisciplinary Function**	A diagnostic and specialty assessment, which is carried out by an experienced multidisciplinary team.
**The Most Responsible Physician (MRP)**	The MRP reviews the care plans of different disciplines working with children on the autism spectrum, ensuring that those plans are integrated, continuous, and coordinated.
**Qualifications**	A preliminary clinical diagnosis using the DSM-5 criteria can be made by child psychologists, psychiatrists, and general pediatricians, who are suitably trained and skilled in the application of those criteria. The diagnostic evaluation team should include a neurodevelopmental or developmental-behavioral pediatrician, child psychiatrist, or pediatric neurologist. A consultant clinical psychologist (Ph.D. or PsyD. holder) acts as a core member of the diagnostic team: A psychologist with doctoral-level training in psychology, which includes psychological testing supervision. Services are overseen by clinical psychologists (Master’s degree level) with experience and expertise in ASD and can seek supervision from consultant clinical psychologists directly, online, or by phone. The behavioral intervention process must be overseen by a professional with a minimum qualification of an M.Sc. in clinical psychology or a related field; it is preferable that she/he holds either a Board Certified Behavior Analyst (BCBA, BCBA-D) or a Behavior Analyst Certification Board (BACB) certificate. The interventionist requires a B.Sc. in psychology or a similar field with courses related to child psychology, disability, child development, and applied behavior analysis (ABA). Occupational therapy (OT) should be provided by a practitioner with an OT B.Sc. or M.Sc., at least five years of clinical experience in pediatric settings, and a license from the Saudi Commission for Health Specialties (SCFHS). Speech and language pathologists (SLPs) with the requisite expertise can contribute to the diagnosis of ASD, normally through joint-working with professionals from other disciplines in an integrated team.

**Table 2:** Recommendations for assessment.

Assessment	
**Medical Assessment**	A medical assessment is recommended for every child on the autism spectrum. Normally, that assessment consists of physical tests (including hearing), screening for dysmorphic features, and a neurocutaneous examination to identify any indicators of tuberous sclerosis. It is also essential to combine observing the child’s current symptoms with a detailed study of his/her record of behavior. That record needs to take into account how the child’s symptoms have developed over time, including assessing how they have impacted his/her functioning within his/her family, among peers, and/or within school or care center settings. The diagnostic process must include formal assessments of the child’s sensory status and his/her adaptive, cognitive, and language capabilities. Both the Social Communication Questionnaire (SCQ) and the Modified Checklist for Autism in Toddlers (M-CHAT) can be used as screening tools for the assessment of ASD. Various questionnaires can be used to review the child’s history of ASD symptoms, including the Social Communication Questionnaire (SCQ), the Social Responsiveness Scale (SRS), and the revised version of the Autism Diagnostic Interview (ADI-R). Similarly, a diagnosis can be confirmed by collecting data through the use of validated tools, such as the second editions of the Childhood Autism Rating Scale (CARS-2) and the Autism Diagnostic Observation Schedule (ADOS-2). The tests referred to above should also incorporate the analysis of the child’s physical development (such as the circumference of their head) compared to standard trajectories, identification of any abnormal differences in body structure or organ size, checking for external evidence of neurocutaneous syndromes, and assessing for neurologic disorders. Assessment of children who have been diagnosed with ASD should include screening for conditions that frequently manifest alongside or have an etiological relationship with ASD. As part of the screening for etiological conditions, all families should be advised to have genetic testing and be provided with the opportunity to have such testing. In cases in which the contributing (i.e., etiological) conditions for a specific developmental issue are unknown, a chromosome microarray should be undertaken. The overall assessment should include evaluating whether the individual has any of the various neurological conditions that commonly afflict children on the autism spectrum, including disorders related to behavior, anxiety, feeding, self-harm, parental attachment, tics, and ADHD. If the assessment reveals the presence of any such disorders, it should be followed by a comprehensive evaluation of those conditions. When it has been identified that the child may have a particular disorder or syndrome, the professional making the assessment should either apply the relevant tests or refer the child to the appropriate specialist.
**Psychological Assessment**	Selecting appropriate methods for conducting this assessment, including but not limited to tests, interviews, observations, surveys, or other data-gathering techniques. Assessing strong points, capabilities, and protective factors that lower the risk of negative outcomes. Establishing a comprehensive developmental and family history. Completing a thorough cognitive assessment, potentially including psychometrics if required. Assessing learning styles, strong points, and any issues impeding learning. Assessing families’ needs.
**Behavioral** **Assessment**	Determining the current level of development by using criterion-referenced and normed-referenced tools (e.g., the Adaptive Behavior Assessment System or the Vineland adaptive behavior scale). Developing an individualized program that focuses on all developmental domains to address current needs.
**Occupational Therapy** **Assessment**	With reference to the DSM-5, assess any issues with the processing of sensory information, including hyper/hyposensitivity. Cognitive and intellectual functioning indicating atypical development. Identify any functional/adaptive challenges that restrict the child’s ability to live fully (e.g., issues relating to sleep, education, personal care, play, safety, or community engagement). Motor development. Academic skills. Food-related problems, including restricted food intake, dietary problems, or mealtime difficulties. Sexuality. Analyze occupational performance to highlight any positive or negative issues related to participation and performance (including evaluating the child’s capabilities and typical patterns of performance alongside the impact of different environmental settings, specific activities, and client factors. All the above should be measured objectively through both non-standardized and standardized assessments).
**Speech–** **Language** **Assessment**	Thoroughly assessing speech and language also includes examining capabilities related to swallowing and eating generally. If required, an assessment of augmentative and alternative communication (AAC) should be undertaken to identify possible benefits related to enhancing the child’s communicative capacities. Identifying participative and activity-related limitations, taking account of the broader health implications of such (e.g., hydrating and having adequate nutrition) and their consequences for the child’s participation in such standard events as meals and family celebrations. Highlighting any issues relating to the individual or their environment that either impede or enhance their consumption of adequate nutrition (e.g., their dietary preferences and familial inputs to implement approaches to safe drinking and eating). Judging the extent to which problems related to feeding and swallowing impact the child’s life and the lives of their family. Such evaluations should take account of the child’s needs, including such issues as facilitating communication through identifying alternative means, augmenting natural speech, or developing communicative practices that encourage more suitable behavior.

**Table 3:** Recommendations for intervention.

Interventions	
**Medical Interventions**	All interventions should be personalized, appropriate to the child’s level of development, suitably intensive, and measured using data that enable the impact of the intervention to be evaluated and its course to be adjusted in line with its overall objective. The prescribing clinician must thoroughly consider both the advantages and disadvantages of using pharmaceuticals to address behavioral issues and should only use such interventions as part of a more holistic approach. The consideration of the advantages and disadvantages of prescription medicine must be based on an understanding of each drug’s required doses, indications and contraindications, potential side effects, relevant drug–drug interactions, and the monitoring required for its usage. Prescription medication can be used to manage a range of conditions and symptoms, including irritability and challenging behaviors. All therapeutic interventions should be informed by the correct diagnosis of any psychiatric issues that manifest alongside ASD. The medicinal intervention pathway should only be taken following the identification of when the behavior commenced and a thorough assessment of factors that trigger it or make it worse.
**Psychological Interventions**	Involve the parents/caregivers, starting with providing them the chance to witness the evaluation process and talk about the detail of their child’s behaviors with the practitioners following that evaluation. Discuss with the parents their child’s strong points, areas for development, and the intervention program that will be delivered so that they can be appropriately engaged in coordinating and advocating for that program.
**Behavioral Intervention**	The ABA principles inform the majority of the intervention models that are based upon evidence of what works. Aspects of the ABA are combined with other principles derived from naturalistic developmental behavioral interventions (NDBIs). Such other NDBI principles include working towards targets for learning and skill acquisition that are developmentally appropriate and delivering interventions within social and environmental settings that are natural. Plans are based on a rigorous comprehensive assessment. Plans should focus on teaching new skills and reducing maladaptive behaviors. Plans should consist of goals that meet the criteria of being specific, measurable, achievable, relevant, and time-bound (i.e., SMART goals). Intensive early intervention programs should be used for children between 2 and 5 years old. Focused interventions and/or parent-mediated interventions may address severe problems and behaviors. Focused interventions might be the model for outpatient services. Family involvement processes should be clearly delineated and systemized in a protocol that defines their level of involvement, training, duration, and expectations. Behavioral interventions are based on the principles of behavior change and aim to decrease difficult behaviors, while teaching alternative behavioral options that the child can employ in challenging situations. There is a range of approaches characterized by the different methods they employ to reach identified objectives. These approaches span from traditional behavioral methods to more modern developmental styles that can be characterized as socially pragmatic. Various factors and preferences must be considered when choosing the right approach. These include those related to the family’s values, resources, and cultural traditions and those related to the child’s linguistic and social development, style of learning, range of behaviors, and communicative requirements.
**Occupational Therapy** **Intervention**	Thirty to sixty minutes of face-to-face interaction with the individual or family/caregivers. Interventions can focus particularly on engaging the child socially, improving his/her occupational performance and adaptive behaviors, or addressing other appropriate familial priorities. Environmental modification to address behavioral, sensory, and functional problems. Liaise with the speech and language pathologist (SLP) regarding child intervention programs and arrange co-intervention sessions to promote social interaction. Sensory and behavioral strategies targeting improving the child’s behavior should be developed and implemented in an integrated way through liaison with their behavioral therapist.
**Speech and Language** **Intervention**	Intervention approaches should focus on building on strengths and reducing challenges related to ASD. They should enable the child to develop or modify their skills and communication strategies in ways that enhance their participation in activities. Changing environmental factors that present barriers to communication while building on those that improve it includes identifying and using suitable augmentative means/accommodations. Similar environmental modifications should be used to encourage appropriate nutritional intake while removing impediments to positive nutritional decisions.

**Table 4:** Recommendations for monitoring.

Monitoring	
**Medical Monitoring**	Systems for monitoring progress should be created for each symptom targeted. The child’s body mass index (BMI) should be measured on standard growth charts and attempts made to pre-emptively modify any identified risks through appropriate advice. All prescribed medications should begin with low doses and be subject to monitoring to identify any side effects. Use of evidence-based guidelines, for example, those produced by the Canadian Alliance for Monitoring Effectiveness and Safety of Antipsychotics in Children (CAMESA), to monitor metabolic side effects [14].
**Clinical Psychology Monitoring**	Regular follow-up assessments of the child’s skills and progress. Monitor progress of the child’s skills and refer for further assessment or services, internal or external, when needed. Follow up on emerging child or family concerns or difficulties. Plan ahead for major transitions for children and families. Monitor and support children and families for emerging comorbid conditions or disorders.
**Behavioral Monitoring**	Reliability and validity of the data are consistently measured on the client’s performance. Evidence of progress report every 6 months for 80–100% of the cases.
**Occupational Therapy Monitoring**	Formal re-evaluation is conducted when the OT identifies (in his/her professional opinion) the emergence of new clinical findings, significant changes in the child’s condition that necessitate further testing and measures, or insufficient reaction to the care plan. Formal re-evaluation is also conducted when extra information is needed pre-discharge.
**Speech and Language Monitoring**	Progress notes are written at intervals that may be stipulated by the facility and report progress on long- and short-term goals.

**Table 5:** Family involvement and family-centered practice.

Recommendations	
**Family Involvement**	Engage the family and other stakeholders in a collaborative approach regarding the referral and assessment processes. Include the family/ caregivers and patient in shared decision making that considers their goals and values. Family should be involved in the generalization of the acquired skills. The child’s needs (and, by extension, family members, etc.) will change over time and differ according to context. Parent-mediated interventions should be considered for children and teenagers on the autism spectrum to promote family engagement, family empowerment, and parental satisfaction.
**Family-Centered Practice**	The goal of family-centered practice is to develop a partnership so that the family/caregivers fully participate in all aspects of the individual. Participation of families/caregivers in services for individuals on the autism spectrum can help to reduce the stress experienced by family members. Support may take different forms at different times. It may include coordinating services for the family/caregivers, organizing resources and providing information, teaching the family/caregivers or other significant communication skills and strategies, providing learning opportunities, and advocating for or with the family.

## Data Availability

All the data for this study will be made available upon reasonable request.
